# Transactions

**Published:** 1882-03

**Authors:** 


### TRANSACTIONS.

The proceedings of the Illinois State Dental Society for the Meeting of 1881, have just been issued by Ramson D. Randolph. It makes a neat and well filled volumn of 170 pages. The papers are, for the most part, excellent, and will well repay thorough reading. The paper on Fractures of the Interior Maxilla, by Dr. T. L. Gilmer, and illustrated by Dr. G. V. Black, is of special interest, being treated very fully and thoroughly, and should receive close attention. His volume should be in the library of every Dentist.

